# Sympathetic nerve activity in stress-induced cardiomyopathy

**DOI:** 10.1007/s10286-012-0162-x

**Published:** 2012-04-11

**Authors:** Yrsa Bergmann Sverrisdóttir, Tomas Schultz, Elmir Omerovic, Mikael Elam

**Affiliations:** 1 Department of Clinical Neurophysiology, Institute of Neuroscience and Physiology, Gothenburg, Sweden; 2 Department of Molecular and Clinical Medicine, Sahlgrenska University Hospital, Gothenburg, Sweden; 3 Department of Physiology, Anatomy and Genetics, University of Oxford, Sherrington Building, Parks Road, Oxford, OX1 3PT UK; 4 Nuffield Department of Surgical Sciences, John Radcliffe Hospital Headington, Oxford, OX3 9DU UK

**Keywords:** Sympathetic nervous system, Cardiomyopathy, Stress, Women

## Abstract

**Purpose:**

To evaluate directly recorded efferent sympathetic nerve traffic in patients with stress-induced cardiomyopathy (SIC).

**Background:**

SIC is a syndrome affecting mostly postmenopausal women following severe emotional stress. Though the precise pathophysiology is not well understood, a catecholamine overstimulation of the myocardium is thought to underlie the pathogenesis.

**Methods:**

Direct recordings of multiunit efferent postganglionic muscle sympathetic nerve activity (MSNA) were obtained from 12 female patients, 5 in the acute (24–48 h) and 7 in the recovery phase (1–6 months), with apical ballooning pattern and 12 healthy matched controls. MSNA was expressed as burst frequency (BF), burst incidence (BI) and relative median burst amplitude (RMBA %). One of the twelve patients in this study was on beta blockade treatment due to a different illness, at time of onset of SIC. All patients were investigated with ongoing medication.

**Results:**

MSNA was lower in patients with SIC as compared to matched controls, but did not differ between the acute and recovery phase of SIC. RMBA %, blood pressure and heart rate did not differ between the groups.

**Conclusion:**

MSNA is shown to be lower in patients with SIC compared to healthy controls, suggesting that sympathetic neuronal outflow is rapidly reduced following the initial phase of SIC. A distension of the ventricular myocardium, due to excessive catecholamine release over the heart in the acute phase, may increase the firing rate of unmyelinated cardiac c-fibre afferents resulting in widespread sympathetic inhibition. Such a mechanism may underlie the lower MSNA reported in our patients.

## Introduction

The maintenance of cardio- and cerebrovascular health is based on a complex relationship between the heart and the brain. While some responses to stress are vital for survival, mental stress has also been claimed to cause cardiovascular disease [1]. The Japanese observation from the early 1990s of a reversible stress-induced cardiomyopathy (SIC), the Takotsubo [2], a peculiar type of left ventricular (LV) dysfunction triggered by an acute strong emotional or physical stressor [3], supports this notion [4]. The syndrome, mostly affecting postmenopausal women, presents signs and symptoms of acute coronary syndrome without evidence of obstructive coronary artery disease. The cause for the evident female predisposition of SIC is unknown, but may be related to gender differences in vulnerability to emotional stress [5] and myocardial sensitivity to catecholamine toxicity [6]. Interestingly, a lower level of estrogen in absence of testosterone in postmenopausal women was recently suggested to explain their greater vulnerability to SIC [7].

Though the definite pathophysiology of SIC remains to be identified, exaggerated sympathetic activation is proposed to be a major contributor to the pathogenesis [8, 9] and forms the basis for treatment of this medical entity. Against this background, the aim of the present study was to directly record and evaluate efferent sympathetic nerve traffic in patients suffering from SIC.

## Methods

### Subjects

Twelve patients with SIC and twelve healthy age, gender and BMI matched controls were recruited for this study. Of the twelve SCI patients, 5 patients were consecutively recruited 24–48 h (acute phase) after SIC onset, while 7 patients (recovery phase, 1–6 months after onset) were recruited from the SCAAR [10] (Swedish Coronary Angiography and Angioplasty Register) and RIKS-HIA [11] (Register of Information and Knowledge about Swedish Heart Intensive Care Admission) registries. SCAAR and RIKS HIA registries contain data on consecutive patients from Swedish hospitals that perform coronary interventions and provide intensive coronary care in Sweden and are sponsored by the Swedish Health Authorities. All patients showed pathologic ECG with evidence of either ST-segment elevation or ST-segment depression and had undergone coronary angiography based on the clinical suspicion of acute coronary syndrome. The following definition for SIC was used: (1) Transient hypokinesis, akinesis or dyskinesis in the left or right ventricular segments and frequently, but not always, a stressful trigger (psychical or physical). (2) The absence of other pathological conditions (e.g. ischemia, myocarditis, toxic damage, tachycardia etc.) that may more credibly explain the regional dysfunction. (3) No or modest elevation in cardiac troponin (i.e. disparity between troponin level and the amount of the dysfunctional myocardium present.

Standard pharmacologic therapy with acetylsalicylic acid, clopidogrel, simvastatin and ramipril was introduced during the first days of hospitalization. Clopidogrel and simvastatin were discontinued after the SIC diagnosis was established while the treatment with acetylsalicylic acid and ramipril was continued. Metoprolol was first introduced after substantial recovery of left ventricular function, which usually occurs within 4–5 days. One of the 12 patients in this study was on beta blockade treatment due to a different illness at time of onset of SIC. This patient did not deviate from the whole cohort of patients in that group. Apart from one patient, none of the patients in this study were on antidepressant or anxiolytic medication.

The body mass score (BMI) was calculated as body weight in kilograms divided by height in meters squared. Systolic (SBP) and diastolic (DBP) blood pressure was measured using a calibrated sphygmomanometer to the nearest 5 mmHg during 15 min of supine rest. Average blood pressure values were derived from three consecutive measurements. Mean arterial pressure (MAP) was calculated by the formula MAP = DBP + (SBP − DBP)/3.

The patients in the recovery group were investigated without withdrawal of ongoing medication recommended for secondary prevention after myocardial infarction and for long-term treatment of heart failure (Beta-blockers, ACE inhibitors, diuretics, nitrates, and digitalis).The study was approved by the local ethical committee.

### Microneurography

Mixed peripheral nerves contain fascicles innervating muscle or skin areas selectively, surrounded by a barrier of connective tissue. The microneurographic technique enables direct recordings of sympathetic action potentials from efferent postganglionic unmyelinated ‘C’ nerve fibres to muscle (MSNA) or skin vascular beds (SSNA) in limb nerves of awake, unanaesthetized humans.

Direct recordings of multiunit postganglionic muscle sympathetic nerve activity (MSNA) were obtained with a tungsten microelectrode inserted into a muscle fascicle of the peroneal nerve posterior to the fibular head. Details of the nerve recording technique and criteria for MSNA have been reported previously [12, 13]. A low-impedance reference electrode was inserted subcutaneously a few centimeters away. When a muscle nerve fascicle had been identified, small electrode adjustments were made until a site was found at which spontaneous, pulse-synchronous bursts of neural activity could be recorded. The original nerve signal was amplified with a gain of 50,000 and fed through a bandpass filter with a band width of 700–2,000 Hz and then through an integrating network with a time constant of 0.1 s, to obtain a mean voltage display of nerve activity. Both the filtered and mean voltage neurograms were stored on a computer (sampling frequency 200 Hz), together with an electrocardiogram (via standard chest leads) and respiratory movements (via a strain gauge attached to a rubber strap around the chest).

Bursts were identified by inspection of the mean voltage neurogram, aided by a computer program developed in the laboratory. MSNA was expressed as burst frequency (bursts/min) and burst incidence (bursts/100 heartbeats). A relative burst amplitude spectrum was obtained and from it a median relative burst amplitude value was extracted and used for statistical analysis [14]. During the microneurographic recording, finger arterial blood pressure was measured non-invasively by the volume-clamp method, the plethysmographic cuff being placed around the middle phalanx of the third finger (Finapres 2300; Ohmeda, Louisville, Kentucky, USA) and heart rate was monitored via ECG-chest electrodes.

### Statistics

Results between the study groups are presented as the mean ± standard error of the mean (SEM) and range. Results within the SIC group are presented as the median and 25th and 75th percentiles due to the small number of subjects in each group. Comparison between groups was performed with the Students t-test for unpaired comparison and within the SCI group comparison was performed with the Mann–Whitney non-parametric *U* test. Correlations were examined by calculating the Pearson linear correlation coefficient. Statistical significance was considered at *p* < 0.05.

## Results

Besides being matched for age and BMI, the study groups did not differ in terms of heart rate and blood pressure level (Table 1).

**Table 1:** Basic characteristics of the study groups

	Patients	Range	Controls	Range	*p* value
Number/gender	12 F		12 F		
Age (years)	66 ± 3.3	48–85	62 ± 2.7	46–74	0.5
BMI (kg/m^2^)	25 ± 1.9	16–34	24 ± 1.2	22–31	0.8
Hemodynamic data					
SBP (mmHg)	113 ± 15	92–140	126 ± 15	92–150	0.6
DBP (mmHg)	64 ± 14	47–80	73 ± 13	57–95	0.2
MAP (mmHg)	77 ± 10	54–92	90 ± 13	72–110	0.4
HR (beats/min)	62 ± 14	45–91	64 ± 7	55–76	0.6
r–r interval (sec)	1.00 ± 0.13	0.65–1.3	0.95 ± 0.06	0.78–1.08	0.5
Microneurography					
MSNA (burst/min)	32 ± 5.0	13**–**68	42 ± 12	24**–**68	0.1
MSNA (burst/100 heartbeats)	49 ± 17	26**–**77	65 ± 12	43**–**90	0.03
MSNA (median amp %)	37 ± 1.6	30**–**47	40 ± 4.6	33**–**46	0.1

Results are presented as mean ± SEM and range

*BMI* body mass index, *HR* heart rate, *MAP* mean arterial pressure

### Muscle sympathetic nerve activity

Muscle sympathetic nerve activity (MSNA) expressed as burst incidence (BI) was significantly lower in patients with SIC compared to matched healthy controls and though not reaching significance, tended to be lower also when expressed as burst frequency (BF) (Table 1). The relative median burst amplitude did not deviate between the groups (Table 1).

Within the SIC group, there was no difference in MSNA BF, BI or relative median burst amplitude between the acute and recovery phase (Table 2).

**Table 2:** Muscle sympathetic nerve activity in patients with SIC in the acute and recovery phase

Variable	SIC acute phase (*n* = 5)	SIC recovery phase (*n* = 7)	*p* value
Age (years)	70 (60–79)	64 (56–72)	0.5
BMI (kg/m^2^)	29 (24–34)	22 (22–24)	0.05
Hemodynamic variables			
SBP (mmHg)	105 (104–122)	113 (98–115)	1.0
DBP (mmHg)	56 (40–67)	60 (57–66)	0.4
MAP (mmHg)	74 (62–80)	78 (77–80)	0.5
HR (beats/min)	77 (51–83)	60 (49–61)	0.4
r–r interval (sec)	0.78 (0.70–1.16)	1.00 (0.98–1.2)	0.4
LVEF (%)	50 (35–60)	60 (57–60)	0.2
Microneurography			
MSNA (bursts/min)	47 (20–50)	25 (17–34)	0.2
MSNA (bursts/100 beats)	57 (39–65)	51 (29–56)	0.3
MSNA (median amp %)	34 (33–42)	39 (32–41)	1.0

Measures are shown as the median and 25th and 75th percentiles

*BMI* body mass index, *HR* heart rate, *LVEF* (%) left ventricular ejection fraction, *MSNA* muscle sympathetic nerve activity

**Fig. 1:**
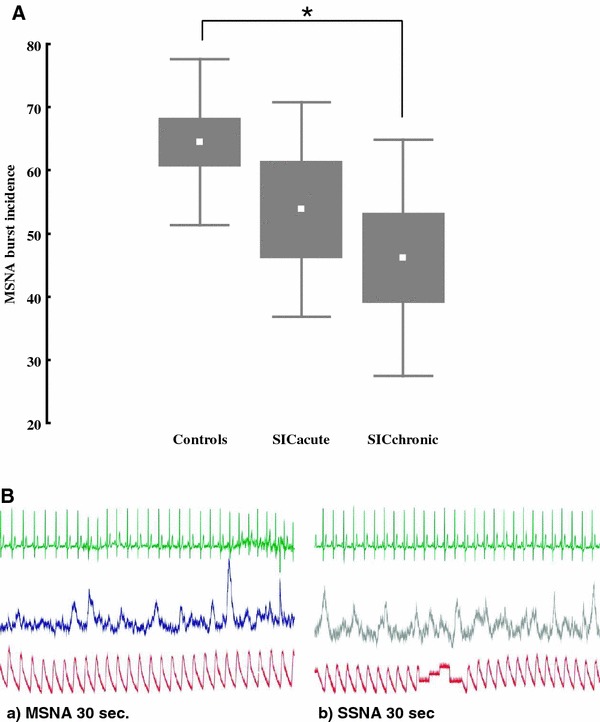
**A** Sympathetic nerve activity, expressed as burst incidence (BI) in 12 patients with SIC (5 in the acute phase and 7 in the recovery phase) and 12 matched controls (*p = 0.03). **B** Examples of microneurographic recordings from a muscle (MSNA) and skin (SSNA) fascicle in a SIC patient in the recovery phase. The figures show a integrated neurogram of MSNA (*a*) and SSNA (*b*) in a 64-year-old female patient with MSNA BF of 17 bursts/min and SSNA BF of 27 bursts/min

MSNA BF and BI did not differ between the control group and the SIC patients in the acute phase (41 vs. 40 b/min, *p* = 0.8 and 63 vs. 54 b/100 hb, *p* = 0.2, respectively), but was significantly lower in the SIC patients in the recovery phase as compared to the control group (41 vs. 26 b/min, *p* = 0.02 and 63 vs. 46 b/100 hb, *p* = 0.03, respectively), (Fig. 1). There was no relation between time from onset of SIC and degree of sympathetic activity.

### Hemodynamic variables

Systolic-, diastolic- and mean arterial blood pressure values did not differ between the groups (Tables 1, 2) and showed no relation to MSNA BF and BI. Heart rate and r–r interval of the ECG was not significantly different between the groups (Tables 1, 2).

Ejection fraction (EF %), as measured by ultrasonic echocardiography, did not deviate between the SIC patients in the acute and recovery phase (Table 2). An apparent inverse relationship between MSNA and EF % was lost when adjusted for age.

## Discussion

The novel finding in this study is that sympathetic outflow to the muscle vascular bed is shown to be lower in patients suffering from stress induced cardiomyopathy as compared to healthy matched controls.

This is in stark contrast to findings in both female and male patients following uncomplicated acute myocardial infarction (AMI), where MSNA was severely augmented as compared to controls, an activation shown to be sustained up to 6-9 months following AMI, despite optimal pharmacological treatment including diuretics, ACE-inhibitors and beta-blockers [15]. In our study, MSNA did not significantly differ between controls and SIC patients in the acute phase, indicating that sympathetic neuronal outflow is rapidly reduced following the initial acute phase of SIC, while it was lower in patients in the recovery phase, which may in part be due to medication, though degree of nerve activity was not related to time from onset of SIC.

Increased plasma catecholamine levels have been reported in SIC and interpreted as evidence for generally increased sympathetic activity, related to the fact that the syndrome is elicited by an acute stressor and associated with an increase in stress-related neuropeptides [8]. Endomyocardial biopsies in some TC patients show contraction band necrosis, a finding consistent with catecholamine mediated cardiotoxicity [9].

Our finding of a reduced MSNA in SIC compared to healthy controls may seem contradictory to previous findings of increased plasma catecholamine, but they may instead reflect different phases of the syndrome. An initial intense sympathetic activation in the acute phase of SIC may cause excessive catecholamine release over the heart and distension of the ventricles. The left ventricular myocardium contains unmyelinated afferents with receptors that are excited both by mechanical and chemical stimuli. It has been proposed that in myocardial distress, these intra-cardiac receptors may function as protective nociceptors, which when activated, can inhibit cardiac contraction and decrease peripheral resistance [16].

A distension of the ventricular myocardium, due to excessive catecholamine release over the heart in the acute phase, could increase the rate of discharge of these unmylellinated cardiac c-fibre afferents, resulting in reflex vagal bradycardia and widespread sympathetic inhibition [17]. Due to such a reflex mechanism, even though recorded in the acute phase, MSNA would not capture the early acute catecholamine release as the reflex would already have ‘kicked in’.

### Sympathetic differentiation

The sympathetic nervous system is a highly differentiated system with a clear distinction in neuronal outflow between sympathetic subdivisions. MSNA and the sympathetic branch supplying the skin vascular bed (SSNA) are the two subdivisions of the sympathetic nervous system accessible with microneurography. MSNA, is mainly involved in hemodynamic regulation and is under strong baroreflex control and correlates well with total body norepinephrine spillover as well as with regional (heart, kidney and subcortical) norepinephrine spillover [18, 19]. In human heart failure though, cardiac adrenergic drive, thought to be of pathogenic importance, has been shown to precede the augmentation in sympathetic outflow to the kidney and muscle vascular beds [20].

SSNA, on the other hand, is mainly involved in thermoregulation and under limited baroreflex control, where only the sudomotor component of SSNA exhibits some cardiac rhythmicity [21]. In thermoneutral conditions, resting SSNA is usually low, exhibiting transient responses to any arousing stimuli. However and despite differences in baroreflex control, it has been speculated that under certain conditions, a common central pacemaker may influence sympathetic outflow to both the heart and the skin [21].

In the present study, recordings of both MSNA and SSNA were obtained in three SIC patients in the recovery phase. Interestingly, all three patients exhibited surprisingly high SSNA, given that the recordings were performed in supine rest at a comfortable room temperature. The SSNA showed clear cardiac rhythmicity suggesting a strong sudomotor contribution, and all three patients exhibited more pulse-synchronous SSNA than MSNA bursts per minute (Fig. 1b). Thus, while MSNA was low in our SIC patients, our observations suggest that SSNA, which is not involved in systemic hemodynamics, may be high, possibly due to enhanced responsiveness to emotional stimuli and may therefore reflect an aroused state. Dissociation in the outflow of these two sympathetic branches has been shown previously in conditions such as aging, heart failure, obesity and essential hypertension, but contrary to SIC, in these conditions MSNA is shown to be high while SSNA is low or unchanged [22].

## Conclusion

Though optimal pharmacological treatment for SIC is not known, most SIC patients are treated according to the general guidelines for treatment of acute myocardial infarction. This therapy includes diuretics, ACE-inhibitors and beta-blockers, medication known in the long-term to inhibit the sympathetic nervous system. Although based on a limited number of patients, our results clearly suggest that sympathetic neuronal outflow is rapidly reduced following the initial acute phase of SIC. Thus, a further understanding of the underlying mechanisms is needed for optimal treatment of the condition.
